# Millennium-Scale Atlantic Multidecadal Oscillation and Soil Moisture Influence on Western Mediterranean Cloudiness

**DOI:** 10.34133/research.0606

**Published:** 2025-02-26

**Authors:** Nazzareno Diodato, Kristina Seftigen, Gianni Bellocchi

**Affiliations:** ^1^ Met European Research Observatory—International Affiliates Program of the University Corporation for Atmospheric Research, 82100 Benevento, Italy.; ^2^ Department of Earth Sciences, University of Gothenburg, 41390 Gothenburg, Sweden.; ^3^ UCA, INRAE, VetAgro Sup, UREP, 63000 Clermont-Ferrand, France.

## Abstract

Understanding long-term historical changes in cloudiness is essential for elucidating Earth’s climate dynamics and variability and its extremes. In this study, we present the first millennial-length reconstruction of the annual total cloud cover (TCC) in the western Mediterranean, covering the period from 969 to 2022 CE. Based on a comprehensive set of hydrological and atmospheric variables, our reconstruction reveals a nuanced pattern of cloudiness evolution over the past millennium. We observe an initial increase in cloudiness until 1600 CE, followed by a substantial decrease in TCC. This shift was driven by a confluence of factors, including the eruption of Mount Tambora in Indonesia in 1815, increased solar forcing, and a positive phase of the Atlantic Multidecadal Oscillation. These complex dynamics have brought modern warming cloud patterns closer to those observed during the medieval period before c. 1250, exceeding the background variability of the Little Ice Age (c. 1250 to 1849). In particular, recent decades have witnessed an unprecedented coupling of intense solar activity, high temperatures, and the lowest cloud cover on record. Our results highlight the importance of inter-oceanic-scale relationships between Atlantic forcing mechanisms and the TCC in shaping future trends in western Mediterranean cloudiness. This study provides valuable insights into the long-term dynamics of cloudiness and its implications for regional climate trends in the western Mediterranean and beyond.

## Introduction

Cloudiness has considerable effects on water cycle patterns and global climate dynamics [1] and is pivotal for Earth’s energy balance, particularly through marine aerosols acting as cloud condensation nuclei [2]. These processes, which occur within the broader context of the water cycle, exert a profound influence on European climate, especially during extreme years [3]. The water cycle, with its intricate processes including clouds, is essential for life [4], a fact that has always been evident to humans [5]. It is impressive how even in ancient times, the Roman architect and engineer Marcus Vitruvius Pollio (c. 80–70 BCE to after c. 15 BCE) understood some of these phenomena in his *Architettura* and approached the water cycle. In the following excerpt, rainfall is apparently assigned the role of landscape, which mostly returns to the atmosphere as clouds (Galiani, p. 180 [6]; translation from [7]:

Hence the winds, wherever they travel, extract from springs, rivers, marshes, and from the sea, when heated by the sun, condensed vapours, which rise and form clouds. These, borne up by the winds when they come against the sides of mountains, from the shock they sustain, as well as from storms, swell and, becoming heavy, break and disperse themselves on the earth.

In this way, in Vitruvius’s description—one of the best of antiquity—clouds play a crucial role in the hydrological cycle, essentially suggesting that evaporation and precipitation are included in a closed cycle: there will be no rain if there are no clouds, and there will no clouds if there is no moisture rising from Earth’s surface [8]. Even today, the response of the cloud cover to changes in the global water cycle remains an outstanding question in future climate projections and in past climate reconstructions. Indeed, as the climate warms, intricate interactions within the climate produce radiative feedback that can either amplify or dampen the overall temperature changes [9].

Current models reflect this complexity by predicting both increases and decreases in cloud cover based on regional conditions and specific climate scenarios. For instance, oceanic warming might lead to reduced cloud cover over land, causing increased solar radiation and local warming, a pattern observed in models of Sahelian drying [10]. Conversely, enhanced terrestrial radiation could stimulate evaporation and cloud formation [11,12]. These contrasting outcomes highlight the critical need for further research to elucidate cloud responses and their broader climatic implications.

**Fig. 1.**
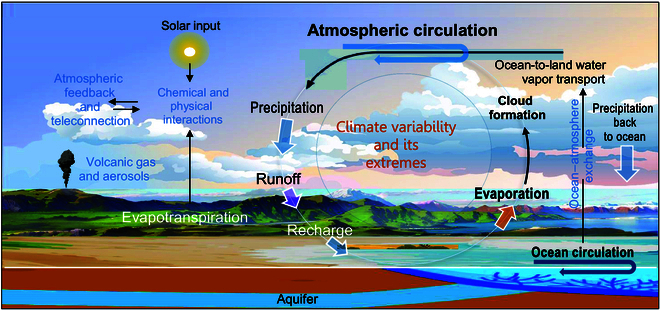
Schematic illustration of Earth’s climate system, including the interplay between the water cycle and clouds. The atmosphere is responsible for transporting water from oceans and land to continents by means of evapotranspiration and winds (atmospheric circulation), influencing cloudiness and precipitation and exerting radiative forcing on the land surface through the radiative effects of clouds, trace gases (such as carbon dioxide and water vapor), and aerosols. Uncertainties in assessing the effects of global-scale perturbations on the climate system are primarily due to a poor understanding of the hydrological cycle and cloud formation. Poorly constrained variability on decadal and longer time scales remains a challenge for training and testing Earth system models. The sky image was arranged from Freepik with the background landscape derived from Google Earth Pro and arranged via VanceAI.

Key uncertainties in these projections stem from the complex interplay between solar forcing, oceanic oscillations, and cloudiness [13], as well as the effects of aerosols and dynamic feedback [14,15]. Clouds, generated by atmospheric circulation, influence both solar radiation absorption and infrared radiation emission while also acting as crucial precipitation sources. Recent research highlights the complex interactions between clouds and aerosols, illustrating how these dynamics affect precipitation characteristics, intensity, and timing [16]. These interactions contribute to intricate feedback mechanisms within the climate system, emphasizing the role of cloud properties in understanding climate variability and change [17]. This interplay creates a complex web of feedback within the climate system [18] (Fig. 1).

**Fig. 2.**
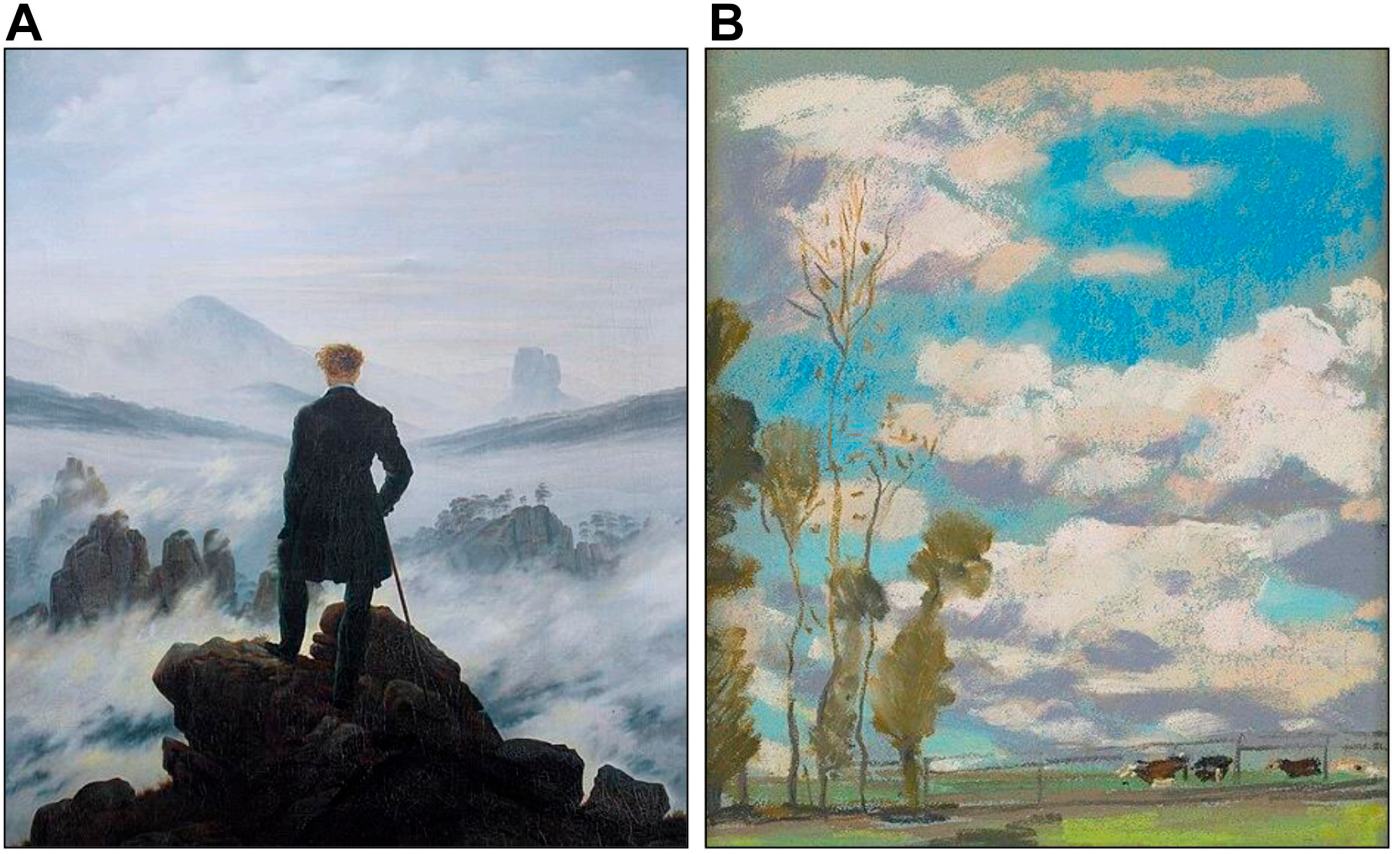
Portraits of specific cloud phenomena. (A) Caspar David Friedrich created one of the most striking paintings of the Romantic period in 1818: *Wanderer above the sea of fog*, in which human figures are a trademark of the German painter, who is best known for depicting them against a starry sky, and the exploration of vast landscapes was widely regarded as a Romantic endeavor throughout most of Europe and the United States [119]. (B) In this picture, Claude Monet depicts a pasture with *Three cows grazing* in 1868 in the distance and a cloudy sky. He highlights the pastureland with a dull green and uses chiaroscuro to fill in the sky with clouds from [120].

The feedback mechanisms of Earth’s climate are critical determinants of the planet’s response to atmospheric forcings [19]. In particular, interactions between clouds and aerosols significantly influence atmospheric deep convection and severe weather events [20]. The scientific exploration of the relationship between aerosols, cloudiness, and cloud formation has been central to meteorology and climate science, providing key insights into these physical processes, as well as inspiring artists. Notably, the Dutch Postimpressionist painter Vincent Willem van Gogh (1853 to 1890), the Norwegian Expressionist painter Edvard Munch (1863 to 1944), the German Romantic landscape painter Caspar David Friedrich (1774 to 1840), and the French Impressionist painter Oscar-Claude Monet (1840 to 1926) (Fig. 2A and B) all beautifully captured these atmospheric processes in their works.

These artistic expressions serve as vivid reminders of the physical processes at play in the atmosphere, underscoring the importance of understanding the scientific phenomena they depict [21].

Interestingly, these 19th-century artistic portrayals align with a period of significant environmental change. During this time, particularly in western Europe, anthropogenic aerosol emissions reached unprecedented levels as a result of the Industrial Revolution [22]. The intricate interplay between anthropogenic aerosols and other natural forcing factors introduces inherent complexities and poses significant challenges, particularly in historical climate modeling and in assessing global climate model performances [23].

This complexity arises from the multiparameter interactions within clouds that influence climate [24] and imply associated feedback mechanisms [25]. Teleconnection patterns, which influence atmospheric variability and extremes over Europe, also play a role in cloud formation and cloudiness [26]. These patterns, associated with large-scale variability modes such as the Atlantic Multidecadal Oscillation (AMO) described by McCarthy et al. [27], which affects long-term North Atlantic sea surface temperatures (SSTs); the North Atlantic Oscillation (NAO), characterized by pressure variations between the Azores High and the Icelandic Low [28]; and the El Niño–Southern Oscillation, which drives periodic changes in equatorial Pacific SSTs [29], are further modulated by small-scale processes arising from the complex physiography of the Mediterranean region [30]. While Zhang et al. [31] primarily investigated NAO effects on cloud phase and radiative forcing over Greenland, their results underscore the broader influence of NAO-related atmospheric circulation changes on cloud behavior in different regions. Rodó and Comín [32] also observed the strong ability of the Southern Oscillation Index, a measure of the El Niño–Southern Oscillation, to predict the interannual rainfall variability over the Iberian Peninsula, potentially influencing cloudiness variability.

Despite the importance of cloudiness in climate studies, there are relatively few long-term records documenting changes at both large [24,33,34] and local scales [35,36]. While satellite-based cloud observations have significantly advanced over the past few decades, their time coverage is generally limited to no more than 30 to 40 years. For example, the International Satellite Cloud Climatology Project [37], one of the pioneering satellite-based cloud observation projects, started in 1983. Data records such as CLARA-A3 and European Space Agency Cloud Climate Change Initiative provide robust datasets spanning about 4 decades [38,39], reflecting the general availability of satellite observations. However, challenges remain, including limited spatial resolution and difficulties in accurately capturing low-lying clouds over complex terrain, which hinder comprehensive analyses of cloud behavior [40]. These constraints, combined with the influence of aerosols and the inherent variability of cloud processes, complicate efforts to develop a more complete understanding of long-term cloud–climate interactions [41].

In the Mediterranean region, the earliest known cloudiness observations date back to the 18th century (1770 to 1784) at the Lamego station in Portugal [42]. Notably, Louis Morin de Saint-Victor (1635 to 1715) documented cloud observations in Paris (France) in 1665 [43], extending our understanding beyond the Mediterranean region. Systematic sunshine and cloudiness records in Spain, dating back to the mid-19th century, often face issues of discontinuity and homogeneity [44].

Analyses of homogenized historical observations reveal a complex evolution of cloud cover over the Iberian Peninsula. Sanchez-Lorenzo et al. [45] found a positive trend in total cloud cover (TCC) in Spain from 1886 to the 1960s, followed by a reversal. Perdigão et al. [46] linked this decadal variability to changes in global radiation over the same period (1964 to 2009). Aparicio et al. [36] further confirmed this trend for the Lisbon region (Portugal), reporting an increase in cloud cover from 1890 to the 1980s, followed by a significant decrease until 2018. The latter tends to correlate with a decline in the atmospheric aerosol load.

Other studies also show a consistent decline in cloud cover over the western Mediterranean region in recent decades. Using data from 35 synoptic stations, Manara et al. [47] found a significant negative trend in TCC over western Italy from 1951 to 2018, consistent with the findings of increased insolation in Mediterranean France from 1931 to 2000 [48] and in eastern Spain from 1985 to 2015 [49]. Indirect evidence from rainfall in western North Africa indicates a similar trend, with a substantial decrease in rainfall between 1971 and 2000, pointing to a decrease in regional cloud cover [50]. Despite these findings, a comprehensive understanding of historical cloudiness and its interaction with climate in the region remains limited due to data scarcity, particularly for both ground-based and satellite observations [51,52]. Further research is needed to bridge gaps and enhance our understanding of climate change in this critical region.

**Fig. 3.**
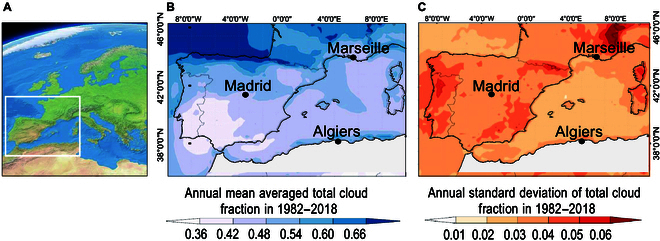
(A) Geographical setting of the western Mediterranean region (white square), arranged from the European Space Agency [121], released under the Creative Commons Attribution-ShareAlike 3.0 IGO (CC BY-SA 3.0 IGO). (B) Annual mean total cloud cover fraction (*TCCf*) and (C) annual standard deviation of *TTCf* over the western Mediterranean provided by European Organisation for the Exploitation of Meteorological Satellites (EUMETSAT) Climate Monitoring Satellite Application Facility (CM-SAF) 0.25° spatial resolution over the period 1982 to 2018, arranged from Climate Explorer.

To generate innovative ideas and provide complementary insights into physical models, adopting a simpler approach—one that streamlines processes and reduce reliance on extensive datasets—can offer additional resources and enhance model accessibility [53]. Here, by using a parsimonious observational approach that relies on simplified inputs, such as prominent forcing indicators and regional climate parameters, we aimed to overcome the limitations of physically based, more complex models, which often struggle with uncertainties arising from incomplete data or inaccuracies in parameterization, especially in regions with complex topography or limited observational coverage. This concept is particularly relevant when considering climatic components, which often act uniformly and consistently across large geographical areas and can signal environmental conditions that influence cloudiness changes. The western Mediterranean is a prime example, where cloudiness patterns are diverse and affected by both large-scale and subregional scale factors (Fig. 3A to C).

In line with this perspective, our study presents the first millennium-long annual reconstruction of the TCC fraction observed from the ground (*TCCf_G_*) for the western Mediterranean, developed from a selected set of hydrological and climatic predictors. The reconstruction model is tuned to the Climatic Research Unit Time Series (CRU TS) v4.06 *TCCf_G_* product, which offers an extended period for robust calibration and validation of the model.

The reconstruction serves as a solid basis to evaluate the cloud fraction over 3 distinct climatic periods: the Medieval Climatic Anomaly (MCA; here 969 to 1249 CE), the Little Ice Age (LIA; here 1250 to 1849 CE), and the Modern Warming Era (MWE; here 1850 to 2022 CE). This effort has produced the longest time series of annual *TCCf_G_*(*H_WM_*) data to date (969 to 2022 CE), providing not only important insights into climate variability over the Common Era but also a key benchmark for climate model simulations over the western Mediterranean and beyond. In addition, the Mediterranean region has been identified as a major “hotspot” for global climate change assessment due to its sensitivity to climatic variations [54], highlighting the relevance of our study in contributing to the understanding of climate change in this critical region.

## Results and Discussion

### Model calibration and validation

TCC information was extracted from the observed CRU TS 4.06 (land) *TCCf_G_* dataset, which has a spatial resolution of 0.50° and covers the period 1935 to present. The CRU TS 4.06 dataset provides gridded observations of various climate variables, including TCC, and is widely used for long-term climate analyses. It applies observational data with reconstruction models to provide consistent climate information over time. For more details on the dataset and its underlying reconstruction methodology, please refer to Materials and Methods.

**Fig. 4.**
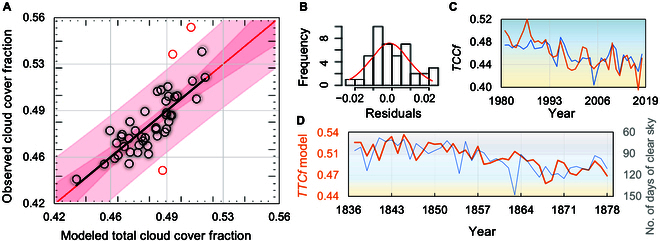
Calibration and validation of the total cloud cover (TCC) fraction model across the western Mediterranean area. (A) Scatterplot between reconstructed (Eq. 1) and observed TCC fractions over the calibration period 1935 to 1980, with the bounds showing 90% (dark-pink-colored area) and 95% (light pink) confidence limits of the reconstruction estimates. (B) The histogram of residuals and its normal fit (red curve). (C) Coevolution between reconstructed (orange line) and observed (blue line) TCC fractions over the validation period 1981 to 2018. (D) Comparison between the reconstructed TCC fraction (orange line) and the independent historical observations of annual clear sky days from Laken et al. [55] (blue line).

Data over the period 1935 to 1980 were used for calibration of the reconstruction model, whereas the 1981 to 2018 period was retained for validation. For the 43-year calibration trial, a strong linear correlation (*y* = *a* + *bx*) was obtained between the actual (*y*) and estimated (*x*) data (*F* test *P* ~ 0.00, *R*^2^ = 0.70), as shown in Fig. 4A.

Only 3 data points (in the years 1970, 1972, and 1979) are outside the 95% prediction bounds, with an intercept of *a* = −0.003 (0.050 standard error) and a slope of *b* = 1.006 (0.103 standard error), as shown in Fig. 4A (light pink band). The standard error of the estimates is 0.009, while the mean absolute error is 0.006. This analysis, conducted on the original (not detrended) data, ensures a comprehensive examination of climatic interactions over both short and long periods, thereby enhancing our understanding of the relationships between the studied variables.

According to the Kolmogorov–Smirnov (*K-S*) test, both samples (actual and predicted) are likely to come from the same distribution (maximum distance between the 2 patterns *DN* = 0.13, *K-S* statistic = 0.63, *P* = 0.83). The model residuals show a skew-free Gaussian distribution (Fig. 4B). There is no evidence of serial autocorrelation in the residuals as the Durbin–Watson (*DW*) statistic (*DW* = 1.56) has a *P* value larger than 0.05 (*P* = 0.051).

The validation of the TCC reconstruction model was carried out from 1981 to 2018. As seen in Fig. 4C, there is a notable consistency between the actual (blue line) and predicted (orange line) cloud cover patterns, with a statistically significant linear relationship between actual and predicted data (*F* test *P* ~ 0.00, *R*^2^ = 0.64). Similarly, the mean absolute error of 0.005 is smaller than the standard error of the estimates (0.007). The Durbin–Watson (*DW*) statistic (*DW* = 1.99; *P* = 0.43) also confirms the absence of serial autocorrelation in the residuals. Similarly, the Kolmogorov–Smirnov test (*DN* = 0.15, *K-S* = 0.47, *P* = 0.98) reveals no statistically significant difference between the 2 distributions, as the *P* value is larger than 0.05.

An additional validation was carried out to evaluate the accuracy of *TTCf* reconstruction. This was accomplished by comparing it with the fully independent historical record from Laken et al. [55], which documents the number of clear-sky days per year (*NDC*). The data were recorded daily in the afternoon by the priest Salvador Bodí y Congrós in eastern Spain, specifically in Carcaixent (39°07′N, 0°26′W), between 1837 and 1878. This validation is critical to test the performance of the model in the preinstrumental era. The *TCCf_G_*(*H_WM_*) estimates and *NDC* per year have a statistically significant relationship, as shown by the coevolution of the 2 variables (Fig. 4D), with an *F* test *P* value of approximately 0.05 and a correlation coefficient (*r*) of 0.61. Again, the *K-S* test shows no statistically significant difference between the 2 distributions (Dn = 0.22, *K-S* statistic = 1.11, *P* = 0.17). These results support the use of our new reconstruction model to derive a robust estimate of western Mediterranean cloud cover during the Common Era. However, it is important to acknowledge potential uncertainties and errors in the reconstruction. These can arise from the relatively coarse temporal resolution of the historical data and inherent observational uncertainties associated with the techniques used. These factors can affect the accuracy and reliability of cloud cover estimates over long periods [56,57].

The parameters defining Eq. 2 are as follows: the scale parameter *A* = 0.01897 transforms the value included in brackets into a cover fraction, while *B* = 0.498 represents the intercept. Additionally, as proportional criteria, the values of *α* = 0.300, *β* = 2.00, *σ* = 0.500, *γ* = −3.00, and *η* = 1.00 serve as modifiers of the cover fraction according to the annual variations of the climatic predictors.

The extended period used for calibration and validation, also covering pre-20th-century climate conditions, allowed for a thorough evaluation of each model predictor variable, namely, the Palmer drought severity index (*PDSI*), storm rainfall index (*SRI*), Atlantic Multidecadal Oscillation (*AMO*), and the 25th percentile of the global temperature anomaly (*GTA*_25*prc*_). Notably, the temperature anomaly term showed the highest *P* value at 0.0070. Thus, it is advisable not to remove any input variable from the model, as each term significantly contributes to the predictive power of the model.

### Spatial correlations among rainfall, *PDSI*, *AMO*, and TCC

**Fig. 5.**
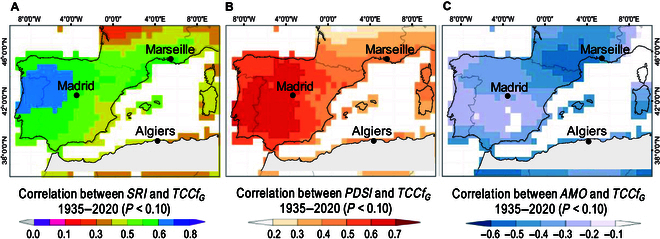
Spatial correlation between climatic factors and cloudiness during the calibration period 1935 to 2020. (A) Correlation coefficient between the reconstructed annual storm rainfall index (*SRI*, 95th percentile of daily rainfall) time series (source: ERA5; time coverage 1950 to now at spatial resolution of 0.25°) over Europe [110] and the observed total cloud fraction (*TCCf_G_*) from Climatic Research Unit Time Series (CRU TS) 4.06 (source: CRU TS Version 4.06; spatial resolution of 0.5°). (B) Correlation between the summer *scPDSI* time series (instrumental, grid developed from University Corporation for Atmospheric Research [UCAR] *scPDSI* [122]; time coverage 1900 to now at a spatial resolution of 0.25°) and *TCCf_G_* field and (C) correlation between the annual Atlantic Multidecadal Oscillation (*AMO*) time series (source: Mann et al. [93]) and the *TCCf_G_* field.

Various approaches have been explored to determine how climatic factors might influence cloud cover at the regional scale. For example, based on the Global Precipitation Climatology Project dataset and using a linear regression approach, long-term trends in convective and low cloud cover were derived from longer-term data on precipitation extremes [58,59], compared to shorter datasets like those from satellite observations. Cox et al. [60] documented a rise in daytime temperatures over large land areas, associating this trend with increased cloud cover, humidity, and precipitation. In China, Wang et al. [23] identified moisture-related variables such as relative humidity and precipitation as primary drivers of cloud cover. In the Mediterranean region, the interannual variability of cloudiness is mainly influenced by changes during the warm season, while the other seasons maintain relatively stable cloud cover levels. Consequently, the warm season emerges as a critical period for understanding interannual cloudiness fluctuations. Notably, storm rainfall aggregated over the year shows a stronger correlation with *TCCf* than annual precipitation alone. This correlation is most pronounced during summer, when cloud cover variability is higher compared to that under the relatively stable winter conditions, primarily influenced by factors that intensified in warmer months, such as precipitation. Consequently, the use of the summer-specific Palmer drought severity index (*PDSI*), reconstructed by Cook et al. [61], becomes pertinent for this analysis. Despite its summer focus, this index effectively captures broader annual climate patterns by incorporating lagged effects and seasonal influences. Thus, by emphasizing the influential April to August period, we can fully understand the dynamics of TCC, taking into account the limited winter variability. In this way, the use of the warm-season *PDSI* better reflects the complex interplay of climatic factors throughout the year. This interplay between precipitation and cloudiness is corroborated by the positive association observed in the western Mediterranean. Furthermore, a significant correlation was established between the storm rainfall index (*SRI*) and the annual *TCCf* CRU TS 4.06 gridded data, with the highest correlation recorded in the westernmost Mediterranean sector, where the corresponding Pearson’s coefficient exceeds 0.55 (Fig. 5A). The reconstructed *SRI*, sourced from the ECMWF (European Centre for Medium-Range Weather Forecasts) Reanalysis v5 (ERA5) dataset, boasts a spatial coverage extending across Europe, enabling the capture of extreme-precipitation events with far-reaching effects on cloudiness patterns. These findings echo those of Richards and Arkin [62] in the eastern Atlantic tropical region, suggesting that expanding spatial and temporal averaging enhances the relationship between predicted rainfall and mean cloud fraction for a given area.

Precipitation is linked to convection from ocean moisture and evapotranspiration by redistributing its transit to the continent, with changes in Mediterranean temperature playing a major role in moisture transfer and higher cloudiness [26]. As a result, changes in temperature affect not only the sensible heat transfer from the ocean and land to the atmosphere but also the evapotranspiration of water vapor at the surface of water and vegetation, resulting in a change in the radiative heating rate of the atmosphere, which in turn affects humidity, clouds, and then precipitation [63]. As a consequence, precipitation positively affects the *PDSI* input data of the *TCCf_G_*(*H_WM_*) model, as an increase or decrease in rainfall typically leads to a rise or fall in soil moisture [64]. *PDSI* is then coupled to the water cycle, with water loss or gain in the soil returning to the atmosphere as an additional source of water vapor and, ultimately, clouds. These interactions are also evident in the Mediterranean region, where *PDSI* affects cloud cover changes throughout the water cycle. Our results highlight the significant association between *PDSI* and *TCCf_G_* throughout the Iberian Peninsula, where the correlation coefficient is greater than 0.50 and is around 0.40 in the rest of the western Mediterranean (Fig. 5B).

Past research has shown that the AMO is a significant, large-scale forcing responsible for interannual *TCCf* variability in the Mediterranean region, with implications for surface air temperature in the northern hemisphere [65]. A negative *AMO*–cloud cover relationship, modulated by SST meridional gradients and storm track activity, was recently corroborated for the North Atlantic by Vaideanu et al. [13]. The findings of Markonis et al. [66] support the hypothesis that atmospheric circulation has a considerable impact on European climate, which can lead to changes in cloudiness. The map in Fig. 5C confirms this interaction by displaying a range of negative correlations, from weak to high, across the western Mediterranean region.

### Historical reconstruction of cloudiness

**Fig. 6.**
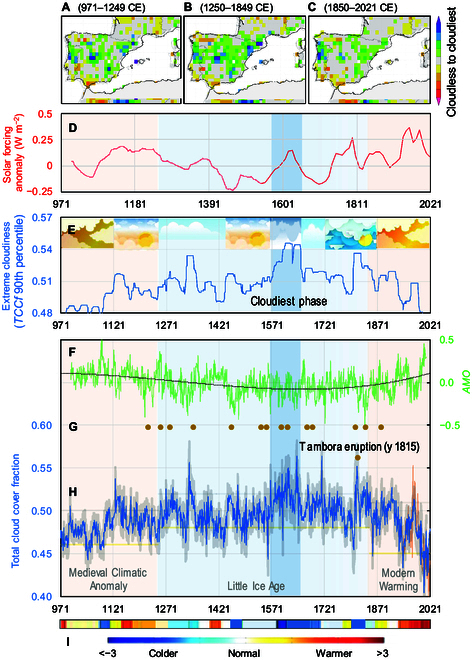
Evolution of cloudiness and climate forcing over the period 971 to 2021 CE for the western Mediterranean. (A) (Map) Water balance indicator (*WBI*) for the period 971 to 1249 CE; (B) (map) *WBI* over 1250 to 1849 CE; (C) (map) *WBI* for the period 1850 to 2021 (A, B, and C, arranged from the 95th percentile *PDSI* data from Cook et al. [61] via Climate Explorer). (D) (Red line) Solar forcing (from Mann et al. [123]); (E) (blue line) multidecadal variability within a moving window 90th percentile of 21 years (cloudier indicator). (F) (Green line) Evolution of the Atlantic Multidecadal Oscillation, with a dark third-order polynomial line (source: Mann et al. [93]). (G) (Brown dots) Stronger volcanic eruptions (from Crowley and Unterman [124]). (H) (Blue line) Annual reconstructed *TCCf_G_*(*H_WM_*) (971 to 2022 CE), with superimposed cloudy sky cover (90th and 10th percentile) thresholds for the Medieval Climate Anomaly (MCA), the Little Ice Age (LIA), and the Modern Warming Era (MWE) period (gray and yellow lines, respectively); the observed *TCCf_G_* at the end of the period is also marked (1935 to 2022, orange line), while the black dots are the volcanic eruption with deposition of sulfate >12 kg km^−2^ (95th percentile of the values of the whole sulfate time series, from Crowley and Unterman [66]). The uncertainty in the reconstructed *TCCf_G_* is quantified as the standard deviation over a 21-year mowing window, with each year centered on the 11th year of the window. (I) (Colored bands) European summer surface air temperature anomalies relative to the 1961 to 1990 climate baseline (arranged from Ljungqvist et al. [125]).

Using Eq. 2, we reconstructed the annual mean of *TCCf_G_*(*H_WM_*) values for the period 971 to 2022 CE (Fig. 6H). The uncertainty in the reconstructed *TCCf_G_* is represented by the standard deviation over a 21-year moving window, capturing temporal variability and providing an uncertainty range for each time point. This method accounts for potential errors in the observational data (predictor uncertainty) and limitations in the reconstruction model (model uncertainty), although further analyses are needed to fully quantify both sources of uncertainty. The 21-year window captures inherent variability in cloud cover due to natural climate oscillations while avoiding excessive noise from short-term fluctuations [67] and aligns with the time scales of major climatic processes [68–70]. Based on this reconstruction, we calculated the differences in the 10th (Fig. 6H, yellow line) and 90th (gray line) percentiles for each of the following periods: the MCA (971 to 1249 CE), the LIA (1250 to 1849 CE), and the last period, the MWE (1850 to 2021 CE). The correlation between *TCCf_G_*(*H_WM_*) and its influencing climatic factors was not stable over time, and the instability was mostly influenced by the expression of Mediterranean water balance processes, which were mediated by temperature and precipitation (Fig. 6A to C). All these conditions, on both large and small spatial scales, probably also played an important role in determining the statistics in the distribution of extreme cloudiness values. For instance, in contrast to a nonsignificant difference in cloudy sky between the MCA, LIA, and MWE periods, there is an important and significant difference in cloudy sky between the recent warming and the LIA.

The apparently not particularly low annual values of *TCCf_G_*(*H_WM_*) during the warm period of the MCA compared to those during the most recent warming should be treated with caution, as historical data during this period are limited and may not fully represent driving information. Basic sources can provide support. For instance, Esper et al. [71] used *Cedrus atlantica* ring widths from the Middle and High Atlas of Morocco to reconstruct late winter to early summer droughts. This reconstruction (included in the broader dataset used by Cook et al. [61] to analyze Old World megadroughts and pluvials during the Common Era) indicates that the MCA was climatically drier than the subsequent LIA. Comparisons with other paleoclimatic records, such as those by Cook et al. [72], highlight similar low-frequency drought patterns. Additionally, Roberts et al. [73] reported that long-term February to June *PDSI* values were above the mean for the period 1400 to 1980 CE and below the mean before that. The LIA was characterized by the longest cold phase in the analyzed period (Fig. 6I, colored bands), accompanied by a predominantly negative AMO phase (Fig. 6F, green line), low solar activity (Fig. 6D, red line), and a concentration of the major volcanic eruptions of the last millennium (sulfate deposition >12 kg km^−2^, Fig. 6G, brown dots). Although the reconstructed precipitation is greater in the first year after the eruption, there is a tendency for the intense cloud cover to last longer. A clustering of eruptions is notably observed with the onset of the LIA, continuing through the cloudiest period. An analogous response pattern is found when using 17 eruptions before 1850 (*scPDSI*), although the response to the only 3 eruptions that occurred during the entire European industrial period since 1850 is readily apparent [74].

Indeed, a pronounced shift toward cloudier conditions was identified after 1400, corresponding to the onset of wetter conditions observed in the Cha2–GP5 δ^18^O records (Chaara and Piste caves) from aragonite stalagmites in northern Morocco caves [75]. This finding is further supported by Fig. 6E, which illustrates the evolution of a cloudy sky indicator (CSI) within a 21-year moving window of the 90th percentile of multidecadal cloudiness. This graph consistently shows CSI values below 0.52, both during the MCA and the MWE. Our reconstruction also indicates reduced cloudiness during the medieval period, similar to that observed in the 20th century before 1990 in the northern hemisphere [76]. Conversely, during the LIA, the CSI exhibited a gradual increase, reaching its maximum (the cloudiest period) between 1550 and 1650 (Fig. 6E, blue band). While Konecky et al. [77] identified a condensation process with lower values during the LIA (1450 to 1850) on a global scale, the Mediterranean experienced a unique and opposing phase. Intensified vorticity associated with Mediterranean cyclogenesis, along with increased convection and moisture from the advection of colder polar or continental air [78], contributed to a cloudier atmosphere compared to that of warmer periods. This aligns with projected Arctic cooling amplification and enhanced precipitation in the Mediterranean [79]. This process is also outlined in Fig. 6F, with the negative phase of the AMO during the LIA, and in the map of Fig. 6B, providing insight into the wetter conditions of the LIA and a resulting cloudier atmosphere compared to those of the medieval and modern warming periods.

This anomalously cloudy phase is evident in the western Mediterranean region, as indicated by the weather information from the 17th-century diary of Rev. Ralph Josselin (1617 to 1683) on climate conditions in England [80]. Josselin observed an increased frequency of easterly or northerly winds, particularly during cold spells, suggesting atypical trends in the jet stream that might have contributed to the unexpectedly stormy weather associated with the European LIA from the mid-16th to the 18th centuries. Historical records reported by Rodrigo and Barriendos [81] also report a high number of wet years across the Iberian Peninsula, mainly in the first half of the 17th century. Overall, from the late LIA, from 1816 onward, the decrease in cloudiness was statistically significant with a decreasing Mann–Kendall test trend (*S* = −10,223, *Z* = 10.40, *P* ~ 0.00), while the enhanced AMO increased significantly with a positive trend (*S* = 6,628, *Z* = 7.27, *P* ~ 0.00).

This phase opposition is concurrently supported by the fact that the AMO begins to transition into the positive phase (Fig. 6F), accompanied by an increase in solar forcing (Fig. 6D) and an incipient rise in temperature (Fig. 6I).

This is consistent with climate models that reflect a strong influence of Atlantic-Multidecadal-Variability-forced SST anomalies on thermodynamic processes [82], associated with a decrease in cloudiness over the Mediterranean [83]. This synchronization suggests that changes in cloudiness in the region also respond to solar forcing, influencing the climate according to multidecadal and millennial solar periodicity. The quasi-1,000-year solar cycle, known as the Eddy cycle [84], and its activity were notably affected during the last millennium by the transition from the MCA and the MWE. A compelling indication of a significant solar–climate interaction is reflected in the words of the American ecologist John Roger Bray (1929 to 2018), who pioneered the concept of a 2,600-year solar-driven climate cycle [85]. Bray [86] claimed that the alignment between past solar activity and climate patterns over centuries and millennia is simply too compelling to overlook. This notion underlines the profound influence of solar variability on Earth’s climate over extended periods. Moreover, a broader perspective emerging from this study of paleoclimatology has led scholars like Rohling et al. [87] to advocate for deeper investigation: “In view of these findings, we call for an in-depth multidisciplinary assessment of the potential for solar modulation of climate on centennial scales” (p. 592). This call for action underlines the importance of integrating different scientific approaches to understand the complexity of solar–climate dynamics. Furthermore, Magny et al. [88] provided additional insights into the centennial-scale climatic events throughout the Holocene: “On a centennial scale, the successive climatic events which punctuated the entire Holocene in the central Mediterranean coincided with cooling events associated with deglacial outbursts in the North Atlantic area and decreases in solar activity during the interval 11700–7000 cal BP, and to a possible combination of NAO-type circulation and solar forcing since ca. 7000 cal BP onwards” (p. 2044). This highlights the complex interplay between solar variability, atmospheric circulation patterns, and regional climate responses on centennial time scales.

The late-19th century culminates in a slight decrease in cloudiness (Fig. 6H, blue line), in tandem with findings reported by Wang et al. [24] for southwest China. Following this period, there was a resurgence of cloudier years, particularly around and after the eruption of the Tambora volcano in Indonesia in 1815. This observation is consistent with research using reanalysis and satellite-based machine-learning techniques, which indicated that aerosols from the eruption enhanced cloudiness by about 10% [23]. Subsequently, cloud cover experienced a continuous decline, punctuated by occasional periods of temporary recovery. Given the observations of the recent decades and for monitoring purposes, it is crucial to track the evolution of the TCC over Spain. Studies from Sanchez-Lorenzo et al. [45,52] reported a declining trend in the TCC that has persisted beyond the 1960s, despite an increasing trend in autumn.

Looking at the tail of the graphs in Fig. 6, it is striking that recent decades exhibit an unprecedented correspondence between intense solar activity (Fig. 6D), high temperature values (band in Fig. 6I), and the lowest cloud cover (Fig. 6H) ever recorded over the last millennium, with the latter falling below the 10th percentile threshold (yellow line in Fig. 6H). This is consistent with the findings of Loeb et al. [89], who discovered that the absorbed solar energy, associated with lower cloud and sea-ice reflections, as well as a decrease in outgoing longwave radiation, outweighed the negative effect of rising global mean temperatures. The authors also showed that both independent satellite and in situ observations yield statistically indistinguishable decadal increases in Earth’s energy imbalance (EEI) from mid-2005 to mid-2019 of 0.50 ± 0.47 W m^−2^ decade^−1^, implying that the increase in absorbed solar radiation is primarily due to natural variations in cloudiness and surface albedo, which have served as the main forcing factors of the flux above the atmosphere over the last 2 decades. EEI, a relatively small difference between global mean solar radiation absorbed and thermal infrared radiation emitted to space, plays a crucial role in understanding Earth’s energy budget and climate dynamics.

## Conclusion

In this study, we conducted a thorough investigation into cloud dynamics in the western Mediterranean, based on multiple historical datasets and contemporary observations. Through model evaluation exercises, we established the reliability of our approach in accurately estimating the total cloud fraction—*TCCfG*(*H_WM_*). Our analysis extended beyond contemporary observations to provide a historical reconstruction spanning the past millennia. This reconstruction revealed significant variability and extremes in cloudiness over the past millennium, with distinct phases corresponding to climatic epochs such as the MCA, the LIA, and the MWE.

Our analysis has uncovered compelling evidence for the complex interplay between solar variability, atmospheric circulation patterns, and regional climate responses on centennial time scales, which is consistent with previous findings that emphasize the profound influence of solar activity on climate dynamics and call for deeper interdisciplinary investigations of solar modulation of climate on centennial time scales. Our results also shed light on contemporary trends in cloudiness, revealing a remarkable correspondence between intense solar activity, high temperatures, and reduced cloudiness in recent decades. This observation is consistent with the broader understanding of the dynamics of Earth’s energy budget and highlights the importance of natural variations in cloudiness and surface albedo in shaping Earth’s climate system. The concept of EEI has emerged as a critical metric for understanding these dynamics, emphasizing the relatively small difference between the global mean absorbed solar radiation and the thermal infrared radiation emitted to space. Our study highlights the importance of incorporating EEI into climate modeling and monitoring efforts, providing insights into the mechanisms driving contemporary climate change.

Overall, this study contributes to a deeper understanding of the complex interactions between solar variability, atmospheric dynamics, and regional climate responses and highlights the need for continued interdisciplinary research to address the challenges of climate change in the Mediterranean and beyond.

## Materials and Methods

### Environmental setting

The western Mediterranean basin plays a significant role on a broader scale by providing the moisture necessary for cloud formation and precipitation. Its distinctive characteristics, such as its coastline, geography, and land–sea interactions, contribute to its significance (Fig. 3A). For example, Fig. 3B depicts the varied geographical pattern of the annual mean total cloud fraction, compiled from European Organisation for the Exploitation of Meteorological Satellites (EUMETSAT) Climate Monitoring Satellite Application Facility (*TTCf_S_*) sequences, from 1982 to 2018. This figure highlights areas of relatively homogeneous cloudiness, such as the southern Iberian Peninsula, across the Mediterranean Sea, and inland northern Africa. In contrast, greater spatial cloud variability and an overall increase in cloud fraction are observed over the northern Iberian Peninsula and southern France, where the maximum *TTCf_S_* values exceed 0.50. Figure 3C, on the other hand, shows the temporal change in *TTCf_S_*, with more pronounced interannual variability over Portugal, southeastern Spain, inland North Africa, the Pyrenees, and easternmost France, where the highest values are reached. These observations underscore the complexity of cloudiness patterns in the region and the need for continued research to fully understand their implications for climate change.

Cloudiness in temperate zones typically arises from atmospheric saturation, primarily induced by frontal low-pressure systems or local adiabatic cooling associated with convection under conditions of atmospheric instability [90]. The Mediterranean basin, characterized by a peripheral and semienclosed sea bordered in the north, south, and east by vast landmasses, exhibits significant influence from large-scale atmospheric patterns on its climate and cloudiness. These factors are modulated by orography and land–sea interactions [91].

### Data sources

In order to assess the historical dynamics of cloud cover over the Mediterranean, with particular emphasis on the western sector, we have compiled a diverse and extensive database of historical and contemporary data sources. This effort led to the development of a reliable collection of annual areal mean cloudiness data, along with additional forcing variables, covering the western Mediterranean from 1935 to 2022. The influential variables included the proxy-based reconstructions of key climate indices: the storm rainfall index (*SRI*), the Palmer drought severity index (*PDSI*), and the Atlantic Multidecadal Oscillation (*AMO*). *SRI* was obtained from the annual flood deposit thickness in lake sediments and served as a proxy for storm rainfall intensity and frequency [92]. The gridded, summer-specific *PDSI*, reconstructed from tree rings from Cook et al. [61], reflects moisture conditions. The annual *AMO* time series is the multiproxy reconstruction from Mann et al. [93], built on a variety of terrestrial and marine proxy records from both hemispheres, including tree rings, ice cores, and coral sediments [94].

To investigate the potential influence of cooler temperature extremes on cloud cover patterns in the Mediterranean, we used the reanalysis-based 25th percentile of the global temperature anomaly (*GTA*_25*prc*_) from Frank et al. [95] in conjunction with the instrumental TCC annual mean fraction observed at the ground level (*TCCf_G_*, CRU TS 4.06 land 0.50°) from Harris et al. [96]. The use of the global temperature anomaly rather than a regional anomaly allows us to assess the influence of large-scale atmospheric circulation patterns on Mediterranean cloud cover. Focusing on the 25th percentile rather than the mean temperature allows us to examine the relationship between cooler conditions and cloud cover, potentially revealing sensitivities not apparent in overall mean trends. This approach is informed by research suggesting that low temperatures are often associated with atmospheric conditions conducive to cloud formation and can provide insights into the region’s sensitivity to temperature variability [97,98]. Long-term cloud cover trends observed in the Mediterranean provide a broader context for understanding the potential impacts of temperature changes on cloudiness in the region [52], while the trend toward drier conditions [99] can further exacerbate the relationship between temperature and cloud cover.

Higher-resolution satellite-derived mean annual fractional cloud cover data (*TTCf_S_*, EUMETSAT 0.25° spatial resolution [100]) for the period 1982 to 2018 were also included. These datasets were accessed via Climate Explorer [101], which not only provides access to various climate datasets but also offers tools for analysis. To ensure that our records were up to date, we used Climate Explorer to access climate data from different sources, enabling us to extend the records until 2022 by retrieving additional data beyond the original end date of the datasets. These sources include instrumental data from the Global Precipitation Climatology Centre (1.0° resolution) for the storm rainfall index, Goddard Institute for Space Studies (World, 2.5° resolution) for the temperature, CRU self-calibrating for *PDSI* (dataset method from Van der Schrier et al. [102]; updates from Osborn et al. [103–105], Lolis et al. [90], Barichivich et al. [106–109], and Bandhauer et al. [110]), and Hadley Centre Sea Surface Temperature for *AMO* [111]. To ensure data homogeneity, we performed linear regressions on overlapping periods between the original datasets and their subsequent updates. This process adjusted the instrumental data to align with the statistical properties (mean and variance) of the historical data, effectively addressing potential discrepancies and ensuring consistency for subsequent analysis. Model calibration was restricted to the historical time series (1935 to 1980) to ensure that adjustments were confined to this period, thereby preserving alignment with contemporary data and achieving overall data homogeneity.

Data analysis was performed using various web-based statistical and graphical software tools, including Statgraphics, Wessa [112], CurveExpert Professional 1.6 for model building and evaluation, and AnClim for time-series analysis and homogenization [113].

### Historical cloudiness model (*TCCf_G_*(*H_WM_*))

As the response of cloudiness to climatic forcing is often influenced by regional-scale climate, changes in *TCCf* in the Mediterranean are influenced by anomalies in some regional climate variables as well as larger-scale teleconnection patterns. These variables include the Palmer drought severity index, the storm rainfall index, the AMO, and the 25th percentile of the global temperature anomaly. These factors guide the development of a multivariate regression model, tailored to the western Mediterranean region, for estimating historical cloudiness (represented as a proportion of total cloudiness), which includes calibration with regional climate data. First, a multivariate linear model was developed, resulting in the response function *TCCf_G_*(*H_WM_*), which represents the annual mean fraction of the TCC. To establish the foundational stage of the *TCCf_G_*(*H_WM_*) model, we used a multiple regression model, denoted as *TCCf_G_*(*MRM*), based on the work by Wilby et al. [114], expressed as

$${\text{TCCf}}_{G}\left(\mathrm{MRM}\right)=\sum_{j=1}^{n}\beta_{j}\times p_{\mathrm{ji}}+\beta_{0}+e_{i}$$ (1)

Here, *p_ji_* are the predictors, *n* is the number of predictors, *β_j_* is a scale parameter, *β*_0_ is a location parameter, and *e_i_* is the modeling error. By solving and extending Eq. 1, we derived the formulation for the historical *TCCf_G_*(*H_WM_*) model with covariate interaction, structured as follows:

$$\begin{array}{c} {\text{TCCf}}_{G}\left(H_{\mathrm{WM}}\right)=\\ A\times \left[\alpha \times \text{PDSI}\times \left(\beta +{\mathrm{SRI}}^{\sigma }\right)-\gamma \times \mathrm{AMO}-\left({\mathrm{GTA}}_{25\mathrm{prc}}+\eta \right)\right]+B \end{array}$$ (2)

where *PDSI* is the Palmer drought severity index, *SRI* is the storm rainfall index, *AMO* is the Atlantic Multidecadal Oscillation, and *GTA*_25*prc*_ is the 25th percentile of the global temperature anomaly. $\text{PDSI}\times \left(\beta +\mathrm{SRI}\right)$ represents an interaction between variables. In this way, the *PDSI* term reflects the positive (negative) shift in soil water deficit as the transition occurs from the wet (dry) year, and then this information is propagated to cloud cover changes via the water cycle element, which is transferred from the surface to cloud formation with the *PDSI* term. However, *SRI* also contributes, implying that changes in storms alone contribute less (Pearson’s correlation coefficient equal to 0.55) compared to the combined influence of *PDSI* and *SRI* on *TCC* (Pearson’ correlation coefficient equal to 0.66).

*AMO* and *TCC* were found to be anticorrelated. The findings of Markonis et al. [66] support that atmospheric circulation has a substantial influence on European climate, contributing to potential oscillations in cloud cover. Long-term shifts in North Atlantic SSTs, including the AMO, are influenced by changes in atmospheric aerosol concentrations. These aerosols interact with the AMO to affect cloud cover over Europe [115–117]. Rising temperatures exert a significant influence on moisture transport and cloud cover [26]. This influence extends beyond the mere transfer of sensible heat between sea, land, and atmosphere, as it also encompasses the evapotranspiration from sea and land surfaces. This process is linked to the variability of the surface thermohaline circulation in the North Atlantic [118]. However, on a millennium-long scale, only global temperature anomaly (GTA) data are available.

**Fig. 7.**
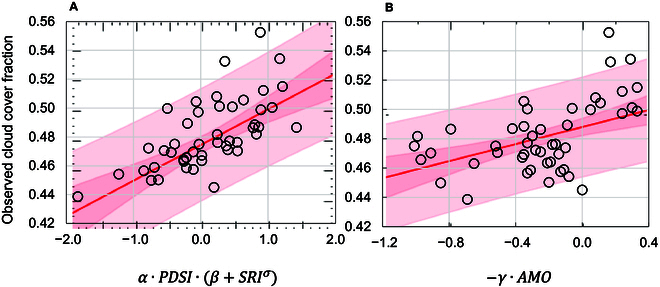
Scatterplots of the correlation between regional- and larger-scale climate factors and cloudiness for the western Mediterranean during the annual-scale calibration period 1935 to 1980. (A) Correlation between the annual water cycle indicator—*WCI* = *α* × *PDSI* × (*β* + *SRI^σ^*)—and the total cloud cover fraction (CRU TS 4.06 land 0.50°) and (B) correlation between *AMO* and total cloud cover fraction. Refer to the text for an explanation of symbols after Eq. 2.

Here, our attention is directed toward the slope regression used to infer long-term variations in cloudiness by analyzing historical patterns in the water cycle, specifically the $\alpha \times \text{PDSI×}\left(\beta +{\text{SIdx}}^{\sigma }\right)$ and *AMO* (γ×AMO) indicators. In particular, Fig. 7A presents the scatterplot of the water cycle indicator (*WCI*) projected against the observed *TCCf_G_*. Notably, only 2 data-point observations fall outside the 95% predicted boundaries (Fig. 7A, light pink band).

Changes in *WCI* have an impact on *TCC*, as variations in soil moisture affect the return of water to the atmosphere through evaporation, which in turn affects cloud formation. The complex interactions present in *WCI* are thus reflected in the changes in cloudiness over the Mediterranean.

Caution is certainly warranted regarding potential confounding factors such as common trends between datasets, but the striking Pearson’s correlation coefficient of 0.66 between *WCI* and *TCCf_G_* suggests the primary role of *WCI* as an indicator of *TCC* change in the western Mediterranean.

Finally, the correlation between *AMO* and *TCC* is presented in Fig. 7B, with a statistically significant relationship (*P* value in the analysis of variance <0.05). In this case, 4 data-point observations are outside the 95% predicted boundaries (Fig. 7B, light pink band).

## Data Availability

All data used in this study are freely available. The graphs presented are original visualisations created by the authors. The full dataset supporting the conclusions of the study is available in the supplementary file (Table S1). TCC information was extracted from the observed CRU TS 4.06 (land) TCCfG dataset (https://crudata.uea.ac.uk/cru/data/hrg/cru_ts_4.06). Datasets were accessed via Climate Explorer (http://climexp.knmi.nl). Data sources include instrumental and reanalysis data from: Global Precipitation Climatology Centre (https://www.dwd.de/EN/ourservices/gpcc/gpcc.html), Goddard Institute for Space Studies (https://psl.noaa.gov/data/gridded/data.gistemp.html), CRU self-calibrating for PDSI (https://crudata.uea.ac.uk/cru/data/drought), and the European Centre for Medium-Range Weather Forecasts (https://confluence.ecmwf.int/display/CKB/The+family+of+ERA5+datasets). Data analysis was performed using various web-based statistical and graphical software tools, including Statgraphics (http://www.statgraphics.com) and CurveExpert Professional 1.6 (https://www.curveexpert.net).
